# An Unconventional Presentation of Multiple Myeloma: Bazex Syndrome

**DOI:** 10.4274/tjh.galenos.2020.2020.0308

**Published:** 2020-11-19

**Authors:** Özlem Kandemir Alibakan, Naciye Demirel, Nihan Nizam, Rafet Eren

**Affiliations:** 1 University of Health Sciences Turkey, Prof. Dr. Cemil Taşçıoğlu Training and Research Hospital, Clinic of Internal Medicine, İstanbul, Turkey; 2 University of Health Sciences Turkey, Prof. Dr. Cemil Taşçıoğlu Training and Research Hospital, Clinic of Hematology, İstanbul, Turkey; 3 İstanbul University İstanbul Faculty of Medicine, Department of Internal Medicine, İstanbul, Turkey

**Keywords:** Bazex syndrome, Acrokeratosis paraneoplastica, Multiple myeloma, Paraneoplastic syndrome

## To the Editor,

Multiple myeloma patients may exhibit signs and symptoms of skin involvement secondary to either malignant cell infiltration or disease-specific treatment. There are also anecdotal reports of paraneoplastic skin diseases including Sweet syndrome, leukocytoclastic vasculitis, and neutrophilic dermatosis associated with multiple myeloma [1]. In this report, we share a patient with a remarkably rare skin presentation of multiple myeloma.

**Figure 1:**
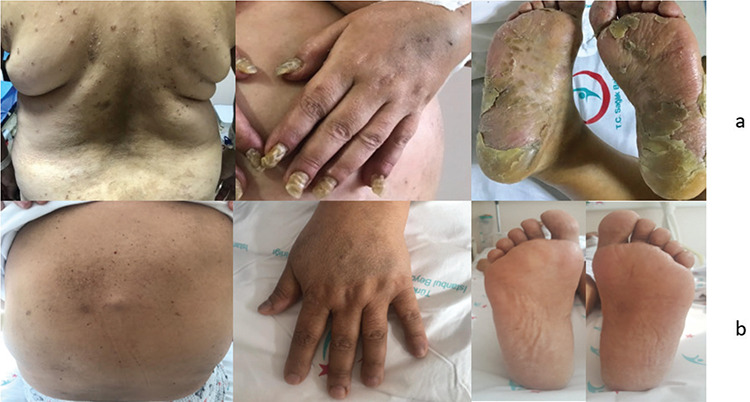
(a) Patient’s lesions at presentation; (b) patient’s lesions after chemotherapy.

A 57-year-old female patient presented to the dermatology clinic with itching, discoloration, hardening, and exfoliation involving the hands, feet, head, and back. Physical examination revealed scalp scaling, hyperkeratosis of all fingernails on both hands and feet, yellowish discoloration, scaly plaques on the palms, thick yellow-gray crusts on the soles, and ichthyosiform appearance of the trunk (Figure 1a). Laboratory evaluation results were as follows: serum creatinine, 1.5 mg/dL; total protein, 14.4 g/dL; albumin, 2.28 g/dL; corrected calcium, 11.74 mg/dL; lactate dehydrogenase, 322 U/L; hemoglobin, 6.9 g/dL; platelets, 103,000/mm^3^; and urine protein/creatinine ratio, 3.29 g/day. A skin lesion biopsy showed Bazex syndrome (acrokeratosis paraneoplastica) and she underwent an investigation to reveal a possible underlying malignancy. Upper and lower GI endoscopy and FDG PET-CT results were normal. She was referred to our clinic for hematologic evaluation. Due to her anemia, elevated creatinine level, and high total protein-to-albumin ratio, plasma cell dyscrasias were suspected and related tests were ordered. Serum immunofixation electrophoresis showed an IgG lambda monoclonal band. Serum free kappa light chain was <6.50 mg/L, free lambda light chain was 475 mg/L, and β2-microglobulin was 14 mg/dL. The patient did not have polyneuropathy, organomegaly, volume overload, endocrinopathy, papilledema, thrombocytosis, or polycythemia and therefore POEMS syndrome was excluded. Bone marrow aspiration and biopsy confirmed plasma cell myeloma (95% infiltration rate, CD38+, CD118+, CD56+, lambda+, kappa-, and Congo stain negative) and FISH was negative for t(14;16), t(4;14), t(11;14), and del(17p13) mutations. The patient was started on bortezomib, cyclophosphamide, and dexamethasone chemotherapy along with topical steroids for the skin lesions as suggested by dermatology. After 4 cycles, her skin lesions were markedly regressed (Figure 1b). Bone marrow aspiration and biopsy showed remission and the patient was referred for autologous stem cell transplantation.

Bazex syndrome is characterized by hyperkeratosis of the acral regions, mostly seen in men over 40 years of age. Lesions are often seen on the nose and ears, and less frequently over acral areas such as the nails, hands, feet, knees, and elbows. Lesions appear as erythematous, violet-purple, and symmetrically distributed papulosquamous plaques [2,3]. Even though its exact etiology is unclear, possible proposed mechanisms include antibodies against the tumor cross-reacting with skin antigens, tumors secreting growth factors such as TGF-α that alter epidermal proliferation, and genetic susceptibility due to HLA-A3 and HLA-B8 [4]. Bazex syndrome may be associated with various malignancies, mostly with squamous cell carcinomas of the head and neck. However, it has also been shown to be associated with many other malignancies, including hematological malignancies [5].

In our literature search, we were able to find only one report of the association of multiple myeloma and Bazex syndrome [6]. This anecdotal association should be considered in patients investigated for Bazex syndrome.
